# Modeling of molecular atomization energies using machine learning

**DOI:** 10.1186/1758-2946-4-S1-P33

**Published:** 2012-05-01

**Authors:** Matthias Rupp, Alexandre Tkatchenko, Klaus-Robert Müller, O Anatole von Lilienfeld

**Affiliations:** 1 Machine Learning Group, Technical University, Berlin, 10587, Germany; 2 Fritz-Haber-Institute, Max-Planck Society, Berlin, 14195, Germany; 3 Argonne National Laboratory, Argonne, Illinois, 60439, USA

## 

Atomization energies are an important measure of chemical stability. Machine learning is used to model atomization energies of a diverse set of organic molecules, based on nuclear charges and atomic positions only [1]. Our scheme maps the problem of solving the molecular time-independent Schrödinger equation onto a non-linear statistical regression problem. Kernel ridge regression [2] models are trained on and compared to reference atomization energies computed using density functional theory (PBE0 [3] approximation to Kohn-Sham level of theory [4,5]). We use a diagonalized matrix representation of molecules based on the inter-nuclear Coulomb repulsion operator in conjunction with a Gaussian kernel. Validation on a set of over 7000 small organic molecules from the GDB database [6] yields mean absolute error of ~10 kcal/mol, while reducing computational effort by several orders of magnitude. Applicability is demonstrated for prediction of binding energy curves using augmentation samples based on physical limits.
